# *Holoptelea integrifolia* (Roxb.) Planch: A Review of Its Ethnobotany, Pharmacology, and Phytochemistry

**DOI:** 10.1155/2014/401213

**Published:** 2014-05-18

**Authors:** Showkat Ahmad Ganie, Surender Singh Yadav

**Affiliations:** Department of Botany, Maharshi Dayanand University, Rohtak, Haryana, India

## Abstract

*Holoptelea integrifolia* (Ulmaceae) is a versatile medicinal plant used in various indigenous systems of medicine for curing routine healthcare maladies. It is traditionally used in the treatment and prevention of several ailments like leprosy, inflammation, rickets, leucoderma, scabies, rheumatism, ringworm, eczema, malaria, intestinal cancer, and chronic wounds. *In vitro* and *in vivo* pharmacological investigations on crude extracts and isolated compounds showed antibacterial, antifungal, analgesic, antioxidant, anti-inflammatory, anthelmintic, antidiabetic, antidiarrhoeal, adaptogenic, anticancer, wound healing, hepatoprotective, larvicidal, antiemetic, CNS depressant, and hypolipidemic activities. Phytochemical analysis showed the presence of terpenoids, sterols, saponins, tannins, proteins, carbohydrates, alkaloids, phenols, flavonoids, glycosides, and quinines. Numerous compounds including Holoptelin-A, Holoptelin-B, friedlin, epifriedlin, **β**-amyrin, stigmasterol, **β**-sitosterol, 1, 4-napthalenedione, betulin, betulinic acid, hexacosanol, and octacosanol have been identified and isolated from the plant species. The results of several studies indicated that *H. integrifolia* may be used as an effective therapeutic remedy in the prevention and treatment of various ailments. However, further studies on chemical constituents and their mechanisms in exhibiting certain biological activities are needed. In addition, study on the toxicity of the crude extracts and the compounds isolated from this plant should be assessed to ensure their eligibility to be used as source of modern medicines.

## 1. Introduction

Plants have been rich source of medicinal agents since time immemorial. They have remained main components of various traditional systems of medicine,* namely*, Ayurveda, Unani, Siddha, Chinese, and so forth. Plant species has remained a good source of anti-infective agents, which are cost-effective and have fewer side effects [1]. Recently the WHO (World Health Organization) estimated that 80% of people worldwide rely on herbal medicines for some aspects. Many developing countries all over the world have intensified their efforts in documenting the ethnomedical data and scientific research on medicinal plants. It is estimated that there are 250,000 to 500,000 species of plants on earth [2]. There are about 47,000 plant species in India, out of which 7,500 plant species are of medicinal value; only 800 plant species are used in the preparation of herbal drugs. So, it is anticipated that plants can provide potential bioactive compounds for the development of new “leads” to combat various diseases. A vast proportion of the available higher plant species have not yet been screened for biologically active compounds; drug discovery from plants should remain an essential component in the search for new medicines. Herbal medicines have recently attracted much attention as alternative medicines useful for treating or preventing lifestyle-related disorders, but relatively very little knowledge is available about their mode of action. There has been a growing interest in the analysis of plant products which has stimulated intense research on their potential health benefits.

## 2. Scope of Review

The review of* H. integrifolia* was primarily needed to bridge the gaps in between traditional uses and* in vitro* pharmacological/biological studies. Previous reviews were based on simple ethnobotanical uses and few pharmacological activities of the species. Hence, an attempt was made to update the complete information on botany, phytochemistry, and pharmacological activities of the species.

**Figure 1:**
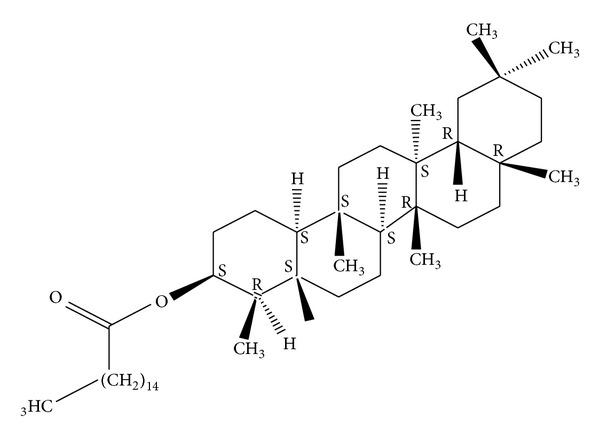
Holoptelin-A.

**Figure 2:**
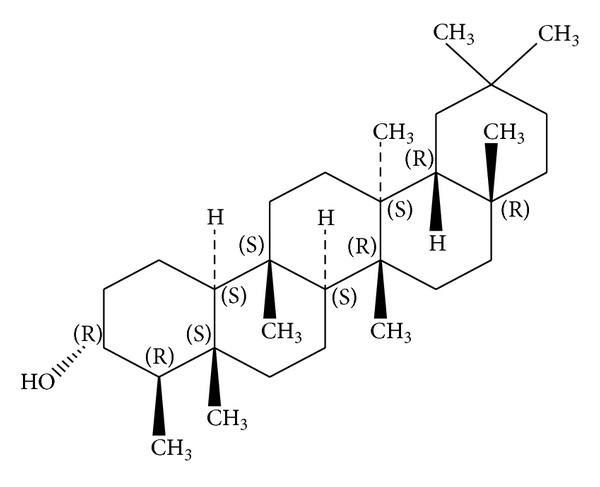
Holoptelin-B.

**Figure 3:**
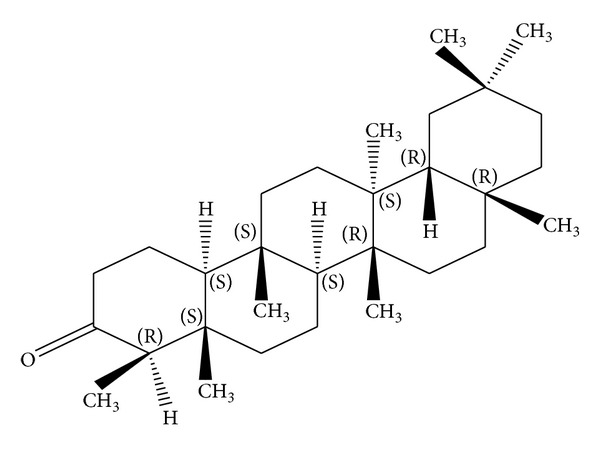
Friedlin.

**Figure 4:**
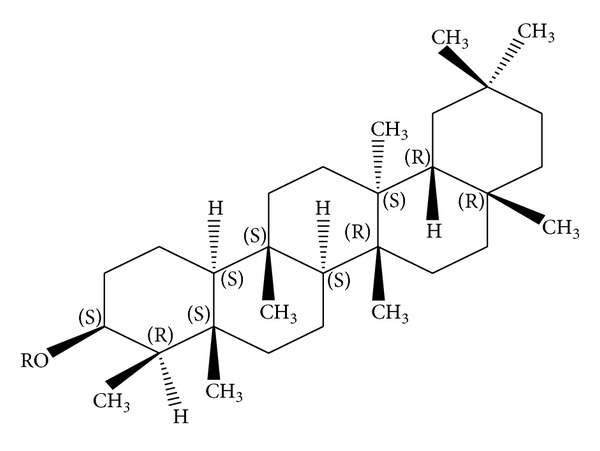
Epifriedlin.

**Figure 5:**
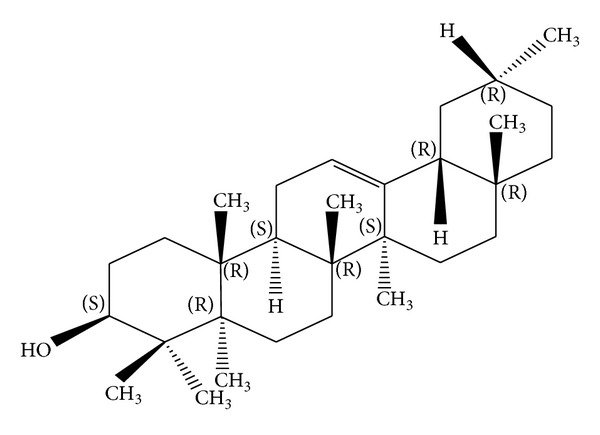
*β*-amyrin.

**Figure 6:**
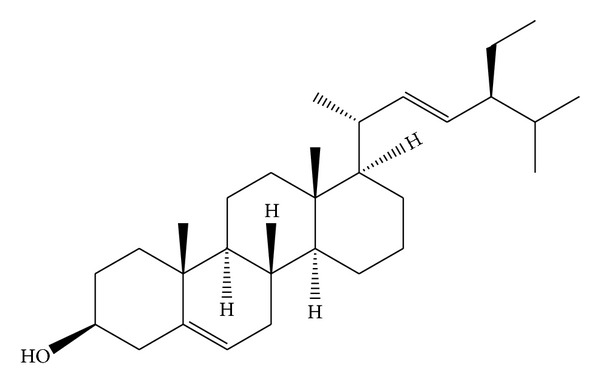
Stigmasterol.

**Figure 7:**
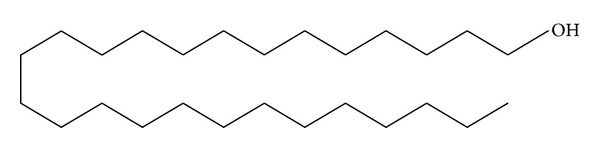
Hexacosanol.

**Figure 8:**
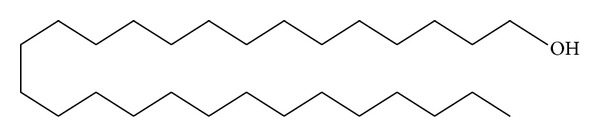
Octacosanol.

**Figure 9:**
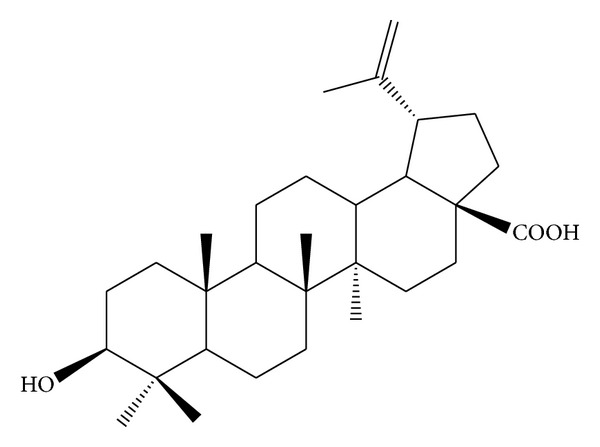
Betulinic acid.

**Figure 10:**
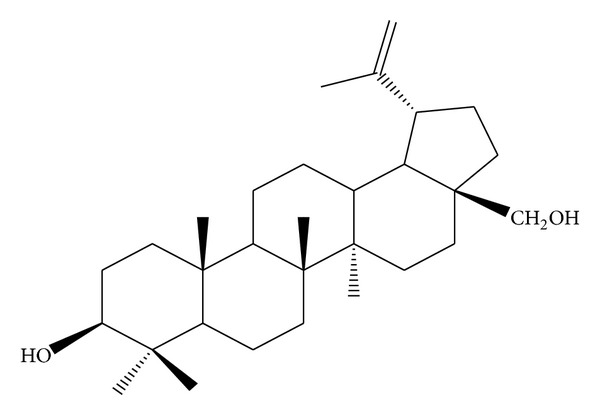
Betulin.

The information provided was taken from different sources like worldwide accepted scientific database Scopus (http://www.scopus.com), Science Direct (http://www.sciencedirect.com), PubMed (http://www.ncbi.nlm.nih.gov/pubmed), Springerlink (http://www.springer.co.in), Google Scholar (http://scholar.google.co.in), and Wiley (http://www.onlinelibrary.wiley.com); thesis; recognized books; abstracts; conference proceedings; and nonimpact and nonindexed journals. The review highlighted the traditional uses of the species in Indian system of medicine (Table 2), secondary metabolites/phytoconstituents isolated from various parts of the plant (Figures 1–10) along with proven biological activity, different biological activities reported on various extracts, and fractions of different plant parts (Table 3). The traditional uses, reported biological/pharmacological activity, isolated compounds, and therapeutic application of* H. integrifolia* might be useful for scientists and researchers to find out new entities responsible for therapeutic activity.

## 3. Botany

### 3.1. Origin and Distribution

The plant species originated from Pacific Islands [3]. It is distributed in temperate and tropical areas of northern hemispheres. The plant is neutralized to the tropical regions of Asia including India, Nepal, Sri Lanka, Indo-China, Cambodia, Laos, Myanmar, Vietnam, Burma, and China [4]. In India it is found in outer Himalayan region from Jammu eastward up to 2000 ft. extending to Assam and Burma, and in southwards from Bengal to Central, Western and South India to dry region of Ceylon.

### 3.2. Taxonomic Status

 Domain: Eukaryota
 Kingdom: Plantae
 Division: Magnoliophyta
 Class: Magnoliopsida
 Order: Urticales
 Family: Ulmaceae
 Genus:* Holoptelea*
 Species:* integrifolia*.

### 3.3. Plant Description

The plant is a large deciduous tree, up to 25 m high. Bark is 6–8 mm thick, whitish-grey, smooth with pubescent branchlets. Leaves are simple, alternate, stipulate, ovate or elliptic-ovate, and acuminate in shape. The bark when cut and leaves when crushed emit an unpleasant odor. Flowers are small, greenish-purple, and polygamous and found in short racemes or axillary fascicles. In male flowers, there are 8 stamens and in bisexual flowers 5 stamens are present. Ovary is superior, unilocular, compressed, and stalked. Style is very short (2.5–4 mm long) with bifid stigma. Fruits are one seeded samara, light brown, obliquely elliptic or orbicular, winged and stalked, indehiscent, and 2.5–3.5 cm long and 1.5–2.5 cm wide. Flowering and fruiting are seen during the months of February-March. Seeds are small, whitish, and kidney shaped.

### 3.4. Vernacular Names

*Holoptelea integrifolia* has many common names depending upon the languages spoken in a particular region. The names used in different languages are presented in Table 1.

### 3.5. Cultivation

The plant is commonly cultivated by the transplantation of nursery-raised seedlings. In the nursery, seedlings are raised by sowing seeds in lines about 12–20 cm apart. Transplantation is done when the seedlings are 10 cm in length at spacing of 22.5 × 22.5 cm. The seedlings are kept in the transplant beds for two years and then planted in the season of monsoon. Fresh seeds are also sown directly at the rate of 2 seeds per stake on the lines of 3 m apart. Continuous watering is required in both cases.

### 3.6. Diseases and Pests

Generally the plant is disease free; however, some pests attack it. Major pests of the tree are the tree borers of the groups Bostrichidae, Buprestidae, Cerambycidae, and Platypodidae, which commonly infest the stem and young leaves.

## 4. Ethnomedicinal Uses

The plant* Holoptelea integrifolia* is used traditionally for the treatment of inflammation, gastritis, dyspepsia, colic, intestinal worms, vomiting, wound healing, leprosy, diabetes, hemorrhoids, dysmenorrhea, and rheumatism [5]. Bark and leaves are used as bitter, astringent, thermogenic, anti-inflammatory, digestive, carminative, laxative, anthelmintic, depurative, repulsive, and urinary astringent [6]. Ethnomedicinally, the leaves and stem bark of* H. integrifolia* are used by tribal people for the treatment of various ailments (Table 2). The mucilaginous bark is boiled and the juice squeezed out and applied to rheumatic swellings [7]. Paste of the stem bark is externally applied to treat the inflammation of lymph glands, ringworm, and scabies. Decoction of the leaves is used to regulate fat metabolism, treat ringworm, eczema, and cutaneous diseases [8]. Stem bark acts as an anti-inflammatory agent specifically for eyes. Stem bark paste is externally applied on forehead of the patient suffering from common fever [9]. Bark and leaf paste of the plant are applied externally on the white patches or leucoderma. Bark boiled in coconut oil and mixed with garlic is applied externally to eczema [10]. For treatment of herpes simplex infection, bark paste is applied over the affected part until it disappears. Bark cut in the shape of a coin is tied on left arm below the shoulder for treatment of malaria [11]. It is also used for the treatment of intestinal cancer [12]. Leaf bud mixed with lime juice is applied externally to affected area for treatment of hair loss by infection [13]. Bark grounded with lemon juice and made into paste is used for weakness [14]. Seeds are used especially on ringworm and dried fruit in polyurea and urinary disorders [15].

## 5. Pharmacology

*H. integrifolia* is known to possess medicinal value in traditional system and represented a wide range of pharmacological properties. Even though several traditional uses of* H. integrifolia* are recognized, a scientific validity and supporting evidence are a prerequisite for commercial exploitation. Table 3 provides an overview on pharmacological properties of* H. integrifolia* extracts as well as compounds isolated from it. In the proceeding text some of the available reports pertaining towards the important pharmacological potential of* H. integrifolia* extracts are being discussed.

### 5.1. Antibacterial Activity

Vinod et al. [16] reported antibacterial effect of hexane, diethyl ether, acetone, and aqueous extracts of leaves of* Holoptelea integrifolia* against lactam resistant strain of* Staphylococcus aureus*. The diethyl ether extract has shown the highest activity and the active principle responsible for the present activity was found to be 1, 4-naphthalenedione. The MIC of the compound was found to be 4 mg/mL.

Padmaa and Durga [17] evaluated petroleum ether, benzene, chloroform, methanolic and aqueous extracts of stem bark of* H*.* integrifolia* for antibacterial activity against* S. aureus, Bacillus subtilis, Escherichia coli,* and* Pseudomonas aeruginosa*. Chloroform extract was found to be most effective against all the test microorganisms. The minimum inhibitory concentration for chloroform extract was found to be 25, 50, 100, and 300 *μ*g/mL against* B. subtilis, S. aureus, P. aeruginosa*, and* E. coli*, respectively.

In the following year, Ahmad et al. [18] studied antibacterial potential of chloroform leaf extract of* Holoptelea integrifolia* against various pathogenic microorganisms,* namely*,* Citrobacter freundii, Micrococcus luteus, Pseudomonas aeruginosa*, and* Pseudomonas fluorescence*. The antibacterial sensitivity was analyzed using disk diffusion method at various concentrations where zone of inhibition was compared with the standard drug cefotaxime. The overall antibacterial activity of* H. integrifolia* was found to be strongest against* C. freundii* followed in descending order by* P. fluorescence, P. aeruginosa*, and* M. luteus*. The minimum inhibitory concentration (MIC) for chloroform leaf extract was found to be 1.562, 3.125, 3.125, and 6.25 mg/mL against* C. freundii, P. fluorescence, P. aeruginosa*, and* M. luteus*, respectively. Another group, Joshi et al. [19], demonstrated antibacterial and antitubercular activity of* H. integrifolia* against different Gram-positive and Gram-negative bacterial strains and* M. tuberculosis* H_37_ RV strain. Alcoholic extract showed moderate to good antibacterial and antitubercular activity.

### 5.2. Antifungal Activity

Besides antibacterial,* H. integrifolia* has a broad antifungal potential [20]. The alcoholic leaf and stem extracts of* H. integrifolia* were studied for antifungal activity against five fungal strains,* namely*,* Candida tropicana, Candida krusei, Candida albicans, Aspergillus niger*, and* Saccharomyces cerevisiae* using the agar well diffusion method. The results indicated that* C. tropicana* (MIC: 39 *μ*g/mL) is most sensitive to MSBE (methanolic stem bark extract) and* S. cerviceae* (MIC: 156.2 *μ*g/mL) and* C. tropicana* (MIC: 156.2 *μ*g/mL) is most and equal sensitive organisms to MLE (methanolic leaf extract). Their MICs ranged from 156.2 to 1250 *μ*g/mL and 39 to 625 *μ*g/mL for MLE and MSBE, respectively. The lowest MMC (minimum microbicidal concentration) observed was 39.04 *μ*g/mL for* C. tropicana* against MSBE.

Sahoo et al. [21] screened the different leaf extracts of* Holoptelea integrifolia* for antifungal activity against the test fungus* Colletotrichum capsici*. The hydroethanolic extract of leaves was fractionated by differential solubility method and only the petroleum ether extract was found to have fungitoxicity. MIC of the active fraction was worked out (200 ppm) and at this concentration it was fungicidal in nature. The active fraction, at MIC, was able to withstand heavy inoculum density, killed the test pathogen in 1.30 hrs. of exposure. It could be more effectively used at any pH between 4 and 9 and may prove to be superior to other commercial fungicides. The active fraction proved to be nonphytotoxic and it stimulated the rate of seed germination and seedling growth of* Capsicum annuum* seeds.

Ishnava et al. [22] evaluated different fractions of* H. integrifolia *leavesfor antifungal activity. The* in vitro* antifungal assay was performed by agar diffusion test and minimum inhibitory concentration (MIC) for hexane, ethyl acetate, and methanolic and aqueous fractions. Among these, ethyl acetate extract exhibited maximum antifungal activity against* Alternaria* sp.,* Aspergillus parasiticus, Aspergillus nidulans, Trichoderma harzianum*, and* Aspergillus flavus* with MIC ranging from 80 to 40 ppm against* Aspergillus nidulans* and* Alternaria* sp., respectively.

### 5.3. Anti-Inflammatory Activity

Anti-inflammatory activity of* H. integrifolia* was reported by Sharma et al. [23]. The activity was analyzed by carrageenan- induced paw edema test in the male wistar strain rats, weighing between 150 and 200 gm. The aqueous extract of* H. integrifolia*, at doses 250 and 500 mg/kg was given to observe % inhibition of paw edema which were comparable with indomethacin (10 mg/kg) used as a reference drug. The extract administered orally produced a significant (*P* < 0.05) dose-dependent inhibition of edema formation.

In another report, Kalpana and Upadhyay [24] investigated the anti-inflammatory properties of ethanolic extract of the leaves of* Holoptelea integrifolia* Planch. A dose of 250 and 500 mg/kg was given to observe percentage inhibition of paw edema which were comparable with indomethacin (10 mg/kg) used as a reference drug. The extract (500 mg/kg) exhibited maximum anti-inflammatory effect (30.01, 21.72, 32.34, and 29.62%) with carrageenan, dextran, histamine, and serotonin method, respectively. A significant percentage inhibition of paw edema by the extract as compared to standard drug suggests its usefulness in acute and chronic anti-inflammatory models.

### 5.4. Antihelminthic Activity

Anthelmintics or antihelminthics are drugs that expel parasitic worms (helminths) from the body, by either stunning or killing them. They may also be called vermifuges (stunning) or vermicides (killing). The first report on antihelminthic activity of* H. integrifolia* was provided by Nadella and Paarakh [25]. The activity of different stem bark extracts was evaluated against* Pheretima posthuma*. Among all the extracts tested, methanolic and aqueous extracts showed better and dose-dependent activity in comparison with reference standard piperazine citrate. Methanolic extract at 80 mg/mL showed shortest time of paralysis (9.0 ± 0.89 min.) and death (35.66 ± 0.81 min.) when compared to piperazine citrate (33.4 and 55.6 min., resp., at 60 mg/mL).

In another study, Kaur et al. [26] comparatively studied the anthelmintic potential of ethanolic and aqueous extract of* H. integrifolia* bark using* Eisenia fetida*. In the bioassay, time of paralysis (*P*) and time of death (*D*) of the worms were determined at various concentrations (10, 25, 50, and 100 mg/mL). The bioassay revealed that the ethanolic and aqueous extract significantly demonstrated paralysis and also caused death of worms especially at higher concentration. The ethanolic extract showed shortest time of paralysis (6.26  ±  0.08 min.) and death (16.73 ± 0.21 min.) at 100 mg/mL. The results were comparable with standard piperazine citrate (2.95 ± 0.09 & 7.10 ± 0.11 min., resp., at 10 mg/mL).

### 5.5. Antioxidant Activity

An antioxidant is a molecule that can terminate chain reactions by removing free radical intermediates and inhibit other oxidation reactions.* Holoptelea integrifolia* possess antioxidants which are responsible for the scavenging activity through various mechanisms. In one study, Saraswathy et al. [27] screened ethanolic crude extract of stem bark of* Holoptelea integrifolia* for its antioxidant activity using *α*-tocopherol as standard antioxidant. The free radical scavenging activity of the extract was evaluated by two different methods, ferric thiocyanate and thiobarbituric acid methods at a concentration of 0.02% and compared with vitamin E. In FTC method, the control showed highest absorbance value (0.83), followed by extract (0.29), standard vitamin E (0.28) on day 5; however, in TBA method, the control had the highest absorbance value (0.35), followed by* H. integrifolia* (0.08) and Vitamin E (0.075). Thus ethanolic extract exhibited significant* in vitro* antioxidant activity by inhibiting the oxidation of linoleic acid in both FTC and TBA methods.

In another study, the methanolic extract of leaves (MLE) and stem bark (MSBE) of* H. integrifolia* was screened for antioxidant activity by 1,1-diphenyl-2-picrylhydrazyl (DPPH) using HPLC method, and total phenolic content was also estimated. MSBE was found to be most potent antioxidant and had more phenolic content than the MLE. The higher phenolic content of MSBE might have contributed to higher antioxidant activity of MSBE [20].

Recently, Srivastava et al. [28] evaluated the* in vitro* antioxidant potentials and phenolic content of aqueous extract of* H. integrifolia* stem bark. The activity was determined by different methods,* namely*, DPPH radical, nitric oxide, superoxide, and reducing power assays. The DPPH radical scavenging activity ranged from 13.14 to 55.17%, comparable to standard ascorbic acid (22.56–93.68%). The IC _50_ value of plant extract for scavenging free radicals was 77.10, 74.95, and 86.78 *μ*g/mL by DPPH, nitric oxide, and superoxide assays, respectively. The total phenolic content was found to be 61.73 ± 0.23 mg GAE/g.

### 5.6. Antidiabetic Activity

Mamatha [29] studied the effect of different extracts,* namely*, ethanolic, petroleum ether, aqueous, and chloroform of* Holoptelea integrifolia* leaves for their antidiabetic activity by using alloxan-induced diabetes models using albino rats. The study indicated that ethanolic, chloroform, and aqueous extracts had more significant (*P* < 0.01) antidiabetic activity (178.3 ± 2.916, 192.7 ± 4.09, and 195.0 ± 2.89, resp.) in acute as well as prolonged treatment (162.7 ± 1.47, 172.9 ± 5.05, and 180.9 ± 2.28, resp.) compared to control (210.7 ± 3.24). The results were comparable with reference standard glibenclamide (170.18 ± 2.58, 159.8 ± 2.53). Petroleum ether extract did not show significant (*P* < 0.05) activity at 7th hour in acute study (207.7 ± 4.42) compared to diabetic control.

Sharma et al. [30] screened methanolic and petroleum ether extract of leaves of* H. integrifolia* for antidiabetic activity. Diabetes in the test animal (male wistar albino rat) was induced by a toxic glucose analogue, Alloxan. The activity was compared with a standardglibenclamide. The petroleum ether extract at 100 and 200 mg/kg and methanolic extract at 200 mg/kg concentration showed significant antidiabetic activity. The activity may be due to presence of steroids and glycosides in the test extracts. These phytocompounds may cause inhibition of ATP-sensitive potassium channels in pancreatic beta cells. This inhibition causes cell membrane depolarization, which causes voltage-dependent calcium channels to open and increase in intracellular calcium in the beta cells which stimulates insulin release.

Recently, Sharma et al. [31] studied antidiabetic activity of stem bark extract of* H. integrifolia* in alloxan-induced diabetic rats. The treatment was continued for three weeks to estimate blood glucose level, body weight, and lipid profile. The blood glucose level decreased gradually in the animals treated with the bark extract (250 and 500 mg/kg b.w.) orally. The prevention of loss of body weight in diabetic animals was found to be significant on day 15 in the animals of both treatment groups.

### 5.7. Antidiarrhoeal Activity

Report on antidiarrheal potential of* Holoptelea integrifolia* was provided by Shrinivas et al. [32]. The ethanolic leaf extract of* H. integrifolia* was studied against diarrhea in mice induced by castor oil and magnesium sulphate. In the experiment, the test extract, at the doses of 250 and 500 mg/kg, reduced the total number of faeces as well as the total number of diarrhoeic faeces in a dose-dependent manner. On the other hand, in the gastrointestinal motility test, the extract at the same doses retarded significantly (*P* < 0.01) the intestinal transit of charcoal meal in mice when compared to the control, atropine sulphate (5 mg/kg).

### 5.8. Adaptogenic Activity

Adaptogens or adaptogenic substances are a new class of metabolic regulators which increases the ability of an organism to adapt to environmental factors and to avoid damage from such factors [33].

Many herbs have been reported to possess adaptogenic agents.* H. integrifolia* is one such a plant which has adaptogenic activity. Kumar et al. [34] reported the adaptogenic activity of ethanolic stem bark extract of* H. integrifolia* in comparison with* Withania somnifera*. The activity was analysed in female albino Wister rats, using forced swimming endurance test and chronic cold restraint stress mode at doses of 250 and 500 mg/kg. Both the doses of test extract were able to increase the swimming endurance when compared with animals who received normal saline.

### 5.9. Anticancer Activity

The effect of* Holoptelea integrifolia* on dalton's ascitic lymphoma in Swiss albino mice was reported by Lakshmi et al. [35]. The ethanolic extract of leaves of* H. integrifoli*a has been tested for anticancer activity at the dose of 250 and 500 mg/kg b.w. The study was conducted to determine the effect of EHI on tumour volume, tumour cell count, viable tumour cell count, mean survival time, and increase in life span. The findings of the study suggested that the crude extract of test plant has potential anticancer activity. The extract increased the life span of DAL treated mice and restored the hematological parameters as compared with the DAL bearing mice in a dose-dependent manner. Soujanya et al. [36] observed anti-breast cancer activity of ethanolic extract of* H. integrifolia* Planch against 7,12-dimethyl benz(a)anthracene induced breast carcinoma in experimental rats. In the study body weight of experimental animals, tumor burden, tumor multiplicity, and tumor volume of breast were determined. The extract administered orally at doses of 250 and 500 mg/kg showed significant (*P* < 0.05, 0.01) dose-dependent inhibition of breast cancer formation. The tumor multiplicity was found to be 1 per group. % inhibition of breast cancer formation by the test extract was almost the same as shown by standard drug tamoxifen.

Recently, Guo et al. [37] observed antineoplastic (anticancer/antitumor) activity of butanol, hexane, ethyl acetate, and chloroform bark extracts of* Holoptelea integrifolia*. The effect was studied on small cell lung cancer, breast, prostate, and colorectal and hepatocellular cancer cell lines. The findings of the study showed that hexane and ethyl acetate extracts had significant cytotoxic effects on breast and prostate cancer cells. Interestingly, hexane extract was selectively over approx. 2-fold more toxic in prostate cancer cells, 2.5-fold in colon cancer cells, and 5-fold in breast cancer cells. Similarly, ethyl acetate extract was selectively approx. 2- to 3-fold more toxic in all the cancer cells compared to non-cancer HEK293 cells. Specifically, the IC_50_ value of only 29.77 ± 1.17 mg/mL of ethyl acetate in prostate cancer cells was over 3-fold highly toxic than HEK293.

### 5.10. Wound Healing Activity

Wound healing is a complex phenomenon, including proliferation of both parenchymal and connective tissue cells, synthesis of ECM (extracellular matrix proteins), remodeling of connective tissue and parenchymal components, and acquisition of wound strength [38].

The wound healing potential of* H. integrifolia* was observed by Reddy et al. [20]. In the study, two different extracts of* H. integrifolia, namely*, methanolic leaf (ML) and methanolic stem bark (MSB) extracts, were analyzed for wound healing potential in albino rats using two wound models, excision wound model, and incision wound model. The findings revealed that, in excision wound model, more than 90% wound healing was recorded in treated groups by 14 days of post-surgery, whereas only 62.99% was observed in the control group. In incision model, higher breaking strengths and higher hydroxyproline content in treated groups suggested higher collagen redeposition than the control group. Besides, the external application of these extracts on the wound prevented the microbes from invading the wound, resulting in the protection of wound against the microbial infections.

### 5.11. Antiulcer Activity

Peptic ulcer disease (PUD) is a serious gastrointestinal disorder that requires a well-targeted therapeutic strategy. Hemamalini et al. [45] reported the antiulcer activity of* H. integrifolia* leaves. The activity of test extract was evaluated using pylorus ligated ulcer model in rats and compared with the standard omeprazole. The methanolic extract at 500 mg/kg per oral dose significantly (*P* < 0.01) reduced the ulcer index (3.43 + 0.06), ulcer formation (69.61%), volume of gastric juice (3.85 + 0.08 mL), free acidity (34.67 + 0.88 Eq/L), and total acidity (69.17 + 1.01 Eq/L) in pylorus ligation- induced ulcer models in rats. The ulcer curative ratio of methanolic extract was almost comparable to that of standard.

### 5.12. Analgesic Activity

An analgesic (informally known as painkiller) is any member of the diverse group of drugs used to relieve pain and to achieve analgesia.

Various medicinal herbs have been reported to have analgesic potential. However, the first study towards the analgesic activity of* Holoptelea integrifolia* was observed by Rizwani et al. [46]. In the study different fractions of* H. Integrifolia* leaves were evaluated for analgesic activity in mice by tail flick method. At the dose of 500 mg/kg the test extracts were found to have a significant (*P* < 0.05) analgesic activity when compared to standard diclofenac sodium (50 mg/kg). Maximum effect was established at 150 min, after drug administration. The maximum analgesic activity was shown by ethanolic extract and followed in descending order by ethyl acetate, *n*-butanol, and aqueous extract.

### 5.13. Hepatoprotective Activity

Hepatoprotection or antihepatotoxicity is the ability to prevent damage to the liver. Recently, Hemamalini and Sathya [47] reported the hepatoprotective activity of methanolic extracts of* Holoptelea integrifolia* against CCl_4_ induced hepatotoxicity. In the study, effect of test extract at a dose of 500 mg/kg on the levels of serum marker enzymes Alanine Transaminase (ALT), Aspartate Transaminase (AST), Alkaline Phosphatase (ALP), and Total Bilirubin (TB) was determined. The study showed that methanolic extract (500 mg/kg) exhibited a significant protective effect by altering the serum levels of AST, ALT, ALP, and total bilirubin. These observations were supported by histopathological studies. Simultaneous treatment of methanolic extracts of* H. integrifolia *with CCl_4_ showed less damage to the hepatic cells as compared to the rats treated with CCl_4_ alone. Histological changes such as steatosis (fatty change, fatty degeneration, or adipose degeneration), inflammatory infiltrations, and perivenular fibrosis were observed in CCl_4_ treated (toxic) control group. The test extract prevented these histological changes, further indicating its hepatoprotective activity.

### 5.14. Mosquito Larvicidal Activity

The first study related to larvicidal activity of* Holoptelea integrifolia* was reported by Singha et al. [48]. The effect of acetone extract from* H. integrifolia* leaves was evaluated on larval mortality of* Culex vishnui* after 24, 48, and 72 h of exposure with five concentrations of crude extract (0.1, 0.2, 0.3, 0.4, and 0.5%). The study showed that the mortality rate of all larval instars at 0.5% concentration was significantly higher (*P* < 0.05) than at 0.1, 0.2, 0.3, and 0.4% concentrations. Highest mortality was observed at 400 ppm concentration of acetone extract. Higher mortality rate was also recorded in 72 h bioassay than those in 24 and 48 h. The results of regression analysis revealed that the mortality rate (*Y*) was positively correlated with the period of exposure (*X*). The log probit analysis (95% confidence level) revealed that LC_50_ values gradually decreased with the exposure period.

### 5.15. Antiemetic Activity

An antiemetic is a drug that is effective against vomiting and nausea. Effect of ethanolic extract of leaves of* H. integrifolia* on cisplatin-induced nausea using a rat model was investigated for antiemetic activity by Shrinivas et al. [49]. Cisplatin at 3 mg/kg dose was selected for testing the antinausea activity of extract. Cisplatin-induced pica decreased significantly when animals were pretreated with* H. integrifolia* extract at doses of 250 and 500 mg/kg.

### 5.16. CNS Depressant Activity

Hemamalini et al. [50]observed the CNS (central nervous system) depressant activity of the methanolic leaf extract of* Holoptelea integrifolia* in Swiss albino mice. A daily dose of 250 mg/kg of extract was administered to the animals for 15 days, after which various CNS experiments such as exploratory behavior and muscle relaxant were recorded and compared with the control animals. The findings revealed that the test extract caused significant reduction in exploratory behavioral pattern in head dip test and reduction in muscle relaxant activity in rota rod and traction tests. These findings confirmed the CNS depressant activity in tested animal models.

### 5.17. Hypolipidemic Activity

Hypolipidemic or antihyperlipidemic agents are lipid-lowering drugs that are used in the treatment of hyperlipidemias. The leaf and bark paste of* Holoptelea integrifolia* is traditionally used for the treatment of obesity in Asian countries. Based on the fact Subhash and Augustine [51] investigated the hypolipidemic effect of* H. integrifolia* and its mechanism in diet-induced obese rat model. In the study body weight, serum lipids, and lecithin: cholesterolacyltransferase (LCAT), apolipoproteins, HMG-CoA reductase, and faecal fat content, were estimated after oral administration. The findings of the study showed that the test extract markedly lowered body weight, serum lipids, HMGR activity, and apo B and increase high-density lipoprotein-cholesterol and apo A1 concentrations. The faecal analysis showed a remarkable increase in faecal lipids, which indicates the ability to inhibit intestinal fat absorption. Presence of a compound 3-(7-ethoxy-4-methyl-2-oxo-2H-chromen-3-yl) propanoate (C1) in the test extract could be responsible for the present activity,which might have inhibited HMGR activity and block intestinal fat absorption.

## 6. Phytochemistry

*Holoptelea integrifolia*, the versatile medicinal plant, is the unique source of various types of compounds having diverse chemical structure. The plant species contains wide range of phytochemicals such as terpenoids, sterols, saponins, tannins, proteins, carbohydrates, and alkaloids [18, 52]. In addition* H. integrifolia* also contains flavonoids, phenols, cardiac glycosides, coumarins, and quinines [53]. The qualitative phytochemical screening of* H. integrifolia* is presented in Table 4. Many compounds were isolated from the plant and proven to be biologically active. Holoptelin-A (Figure 1), Holoptelin-B (Figure 2), friedlin (Figure 3), epifriedlin (Figure 4), 2-aminonaphthoquinone, *β*-sitosterol, *β*-D-glucose, *β*-amyrin (Figure 5), stigmasterol (Figure 6), and hederagenin have been isolated from heart wood and bark while hexacosanol (Figure 7), octacosanol (Figure 8), *β*-sitosterol, and *α*-amyrin were isolated from leaves [28]. Sadasivan et al. [16] isolated an antibacterial compound 1, 4-naphthalenedione from diethyl ether leaf extract of* H. integrifolia*. Recently, Ahmad et al. [54] isolated two medicinal pentacyclic triterpenoids, betulinic acid (3*β*-Hydroxy- lup-20(29)-en-28-oic acid) (Figure 9), and betulin (Lup-20(29)-ene-3*β*, 28-diol) (Figure 10) from methanolic bark extract. These isolated compounds were identified as betulinic acid and botulin, respectively, on the basis of detailed spectroscopic analysis and their comparisons with the data reported in the chemical literature for these components. The distinguishable position in 13C-NMR of betulin was found at C-17 = 47.8 ppm and C-28 = 60.8 & 60.6 whereas in the betulinic acid the value of the same carbon signals depicted in the spectrum was at 56.3 & 56.26 and 180.5 & 179.62 due to the attachment of CH_2_OH and COOH, respectively. Betulinic acid has valuable biological potential, such as inhibitors of HIV-1 entry, HIV-protease, or of reverse transcriptase (RT), whereas betulin had significant anticancer effect on adenocarcinoma, cervix carcinoma, hepatoma, and breast cancer.

## 7. Conclusion

It is evident from the available literature that* Holoptelea integrifolia* leaves are the most investigated part of the plant. Leaf and stem bark has been a promising agent for the treatment of skin diseases like leucoderma, scabies, ringworm, and eczema. The biological studies such as antimicrobial, antihelminthic, antidiabetic, anti-inflammatory, and antioxidant activities were noticeable in crude extracts of various parts of the plant. In parallel to above observations pure compound 1, 4-napthalenedione was marked for antibacterial activity against *β*-lactam resistant strain of* Staphylococcus aureus*. The detailed study of pharmacology, bioassay guided isolation of active principles, and mechanism of action may help to understand the relation between pharmacological effects and traditional uses of* H. integrifolia*. There are many areas to work in this plant for its full recognition. Factors such as geographical and seasonal variation play an important role in the authentication of the chemical constituents responsible for the activity which also can be an area of interest. Thus, it is mandatory to fill the huge gap of insufficient knowledge and awareness among pharmacologists as well as researchers to hold the position of this plant in providing better medicinal values to the society. This can be fulfilled only by generating interest among research community through writing reviews and carrying out research on different aspects of this plant species.

## Figures and Tables

**Table 1:** Vernacular names of *Holoptelea integrifolia*.

Language	Vernacular name
Hindi	Papri, Chilbil, Kanju, Banchilla, Bawal, Poothigam, Dhamna, Begana, Chirabil
English	Indian elm, Jungle cork tree, Monkey biscuit tree, Indian beech tree
Sanskrit	Chirivilva, Pootikaranja, Udakirya, Hasthivaruni, Markati, Vayasi, Karabhanji
Malayalam	Aavil, Njettaval, Aval
Punjabi	Rajain, Khulen, Arjan
Telugu	Nemilinara, Nali, Thapasi, Nemali, Pedanevili
Kannada	Kaladri, Nilavahi, Rahubija, Thavasai, Rasbija
Tamil	Aya, Ayil, Kanci, Vellaya, Avil, Pattai
Bengali	Nata karanja
Marathi	Ainasadada, Vavala, Vavli, Papra, Bawal
Oriya	Dhauranjan, Turuda, Karanja
Gujarati	Charal, Charel, Kanjo, Waola, Chirbil, Chirmil
Konkani	Vamvlo
Burmese	Myaukseik, Pyaukseik
Nepali	Sano pangro
Siddha	Iya

**Table 2:** Ethnomedicinal profile of *Holoptelea integrifolia*.

Disease/disorder/indication	Part used/mode of application	Reference
Leprosy, boils, inflammation, skin disease, and scorpion sting	Leaf is boiled in water and water bath is given	[39]
Rickets	Young leaf is applied on back bone and tied for 1 hr.	[40]
Headache	Bark is made into a paste and applied	[41]
Chronic wound	Bark powder is applied	[42]
Leucoderma, leprosy, scabies, and other skin diseases	Leaf and bark	[43]
Uncontrolled bleeding, fresh wound	Seeds are applied externally in the form of poultice on the injured part	[44]
Rheumatism	Mucilaginous bark is boiled; juice is squeezed out and applied to swellings	[7]
Ringworm, eczema, and cutaneous diseases	Decoction of the leaves	[8]
Common fever	Stem bark paste is externally applied on forehead	[9]
Eczema	Bark boiled in coconut oil and mixed with garlic is applied	[10]
Malaria	Bark cut and tied on an arm	[11]
Intestinal cancer	Bark	[12]
Herpes infection	Leaf bud mixed with lime juice is applied externally to affected area	[13]
Weakness	Bark grounded with lemon juice and made into paste is given	[14]
Polyurea and other urinary disorders	Dried fruit is used	[15]

**Table 3:** Pharmacological activities of various extracts of *Holoptelea integrifolia*.

Activity	Plant part	Extract	Experimental procedures/models	References
Antibacterial	Leaves	Hexane, diethyl ether, acetone and aqueous	MIC (minimum inhibitory concentration)	[16]
	Stem bark	Pet. Ether, benzene, chloroform, methanolic and aqueous	MIC (minimum inhibitory concentration)	[17]
	Leaves	Chloroform	Disk diffusion assay	[18]

Antifungal	Leaf and stem	Methanolic	Agar well diffusion method	[20]
	Leaves	Hydroethanolic	MIC (minimum inhibitory concentration)	[21]
	Leaves	Hexane, ethyl acetate, methanolic, and aqueous	Agar diffusion test and MIC (minimum inhibitory concentration)	[22]

Anti-inflammatory	Leaves	Aqueous	Carrageenan-induced paw edema test	[23]
	Leaves	Ethanolic	Carrageenan-induced paw edema, dextran-induced paw edema, histamine-induced paw edema, serotonin-induced paw edema, and cotton pellet-induced granuloma tests	[24]

Anthelmintic	Stem bark	Benzene, chloroform, methanolic, aqueous, pet. ether	Dash's method	[25]
	Bark	Ethanolic and aqueous	Time of paralysis and death assay	[26]

Antioxidant	Stem bark	Ethanolic	FTC (ferric thiocyanate) and TBA (thiobarbituric acid) methods	[27]
	Leaves	Methanolic	1,1,diphenyl-2-picrylhydrazyl (DPPH) method	[20]
	Stem bark	Aqueous	DPPH radical, nitric oxide, superoxide, and reducing power assays	[28]

Antidiabetic	Leaves	Ethanolic, chloroform, pt. ether and aqueous	Alloxan-induced diabetes model	[29]
	Leaves	Methanolic and pet. ether	Alloxan-induced diabetes model	[30]

Antidiarrhoeal	Leaves	Ethanolic	Castor oil and magnesium sulphate-induced diarrhea mice model	[32]

Adaptogenic	Stem bark	Ethanolic	Forced swimming endurance test and chronic cold restraint stress mode	[34]

Anticancer	Leaves	Ethanolic	Dolton's ascetic lymphoma (DAL) model	[35]
	Bark	Ethanolic and pet. ether	DMBA- (dimethyl benz(a) anthracene-) induced breast carcinoma assay	[36]
	Bark	Butanol, hexane, ethyl acetate and chloroform	MTT 3-(4,5-dimethylthiazole-2yl)-2,5-biphenyl-tetrazolium bromide assay	[37]

Wound healing	Stem bark and leaves	Methanolic	Excision and incision wound model	[20]

Antiulcer	Leaves	Methanolic	Pylorus ligated ulcer model	[45]

Analgesic	Leaves	Ethanolic, ethyl acetate, n-butanol and aqueous	Tail flock method	[46]

Hepatoprotective	Leaves	Methanolic	Paracetamol-induced hepatotoxicity assay	[47]

Larvicidal	Leaves	Acetone	Standard WHO procedure	[48]

Antiemetic	Leaves	Ethanolic	Cisplatin-induced nausea model	[49]

CNS depressant	Leaves	Methanolic	Head dip, Rota rod, and traction tests	[50]

Hypolipidemic	Leaves and bark	Hydroalcoholic	HMG-CoA reductase and LCAT (Lecithin: cholesterol-acyl transferase) assays	[51]

**Table 4:** Qualitative phytochemical screening of *Holoptelea integrifolia*.

Solvent	Plant part	Reducing sugar	Protein	Phenol	Alkaloid	Steroid	Triterpenoid	Flavones	Tannin	Saponins	Reference
Pt. Ether	Leaf	+	+	−	−	+	−	+	+	−	[8]
	Stem	−	−	−	−	+	−	−	+	−	
Benzene	Leaf	−	−	−	−	+	−	+	+	−	
	Stem	−	+	−	−	+	−	+	+	−	
Chloroform	Leaf	−	+	−	−	+	−	−	−	−	
	Stem	−	+	+	−	+	−	+	−	−	
Methanol	Leaf	−	+	−	+	+	−	−	−	−	
	Stem	−	+	+	+	+	+	+	+	−	
D. Water	Leaf	−	−	+	−	−	+	−	+	−	
	Stem	−	−	−	−	−	+	−	+	−	
Acetone	Leaf	+	+	+	+	+	−	+	+	+	[18]

(+): present and (−): absent.
